# Real-World Evidence on the Effect of Missing an Oral Contraceptive Dose: Analysis of Internet Search Engine Queries

**DOI:** 10.2196/20632

**Published:** 2020-09-15

**Authors:** Irit Hochberg, Sharon Orshalimy, Elad Yom-Tov

**Affiliations:** 1 Institute of Endocrinology, Diabetes and Metabolism Rambam Health Care Campus Haifa Israel; 2 Bruce Rappaport Faculty of Medicine Technion – Israel Institute of Technology Haifa Israel; 3 School of Public Health Ben Gurion University Beer Sheva Israel; 4 Microsoft Research Herzeliya Israel; 5 Faculty of Industrial Engineering and Management Technion – Israel Institute of Technology Haifa Israel

**Keywords:** search engines, birth control, abortion, miscarriage

## Abstract

**Background:**

Oral contraceptives (OCs) are a unique chronic medication with which a memory slip may result in a threat that could change a person’s life course. Subjective concerns of missed OC doses among women have been addressed infrequently. Anonymized queries to internet search engines provide unique access to concerns and information gaps faced by a large number of internet users.

**Objective:**

We aimed to quantitate the frequency of queries by women seeking information in an internet search engine, after missing one or more doses of an OC; their further queries on emergency contraception, abortion, and miscarriage; and their rate of reporting a pregnancy timed to the cycle of missing an OC.

**Methods:**

We extracted all English-language queries submitted to Bing in the United States during 2018, which mentioned a missed OC and subsequent queries of the same users on miscarriage, abortion, emergency contraceptives, and week of pregnancy.

**Results:**

We identified 26,395 Bing users in the United States who queried about missing OC pills and the fraction that further queried about miscarriage, abortion, emergency contraceptive, and week of pregnancy. Users under the age of 30 years who asked about forgetting an OC dose were more likely to ask about abortion (1.5 times) and emergency contraception (1.7 times) (*P*<.001 for both), while users at ages of 30-34 years were more likely to query about pregnancy (2.1 times) and miscarriage (5.4 times) (*P*<.001 for both).

**Conclusions:**

Our data indicate that many women missing a dose of OC might not have received sufficient information from their health care providers or chose to obtain it online. Queries about abortion and miscarriage peaking in the subsequent days indicate a common worry of possible pregnancy. These results reinforce the importance of providing comprehensive written information on missed pills when prescribing an OC.

## Introduction

The World Health Organization (WHO) recognized contraceptives and family planning methods as key elements for fulfilling human rights and promoting women's autonomy and wellbeing [1]. Therefore, a wide range of contraception options are included in their Model List of Essential Medicines. Contraceptive access, choice, and knowledge as well as correct contraceptive use are key elements in increasing compliance to and efficacy of contraception and decreasing the number of unintended pregnancies [2,3].

A main component of the WHO guidelines for contraceptive use refers to the quality of contraceptive counseling and delivering correct information before choosing a method of contraception [4]. Misuse of contraceptives and discontinuation are associated with a lack of knowledge about contraceptives, individual lifestyles, social structure, age, education, class, ethnicity, and race [5-8]. Oral contraceptives (OCs) are the most commonly used contraception in the United States [9], and optimal use over a year prevents 99.5% pregnancies, while nonoptimal use has a failure rate of >7% over a year [10]. Age is an important demographic characteristic for OC misuse, as young women and adolescents report forgetting a pill more often than others [5,11-13] and have a much higher rate of failure of oral contraception compared to older women [14]. Multiple studies have shown that approximately 1 of 2 young users misses 1 or more OC pills each month [15-18]. It is important to give clear instructions on the appropriate measures to prevent pregnancy after a missed dose while providing contraception counseling to women, according to their lifestyles, sexual partner status, and health condition [5,19].

OCs are a unique chronic medication where a slip of memory may be experienced as a threat that could change the life course of a person. Subjective experiences and concerns in women missing doses of OCs have been addressed infrequently, but there is evidence that this event leads to stress and affects women’s well-being and ability to function at work [20].

Past research shows that young people frequently prefer to search for sexual health advice online, over turning to a health care provider they might have seen in person months before, due to the accessibility and privacy afforded by online search [21,22]. Anonymized queries to internet search engines provide a unique access to the incidence of concerns and information gaps in a large number of internet users [23-25]. Further queries by the same users can shed light on their attitudes, behaviors, and health consequences in the period following the query [26]. In this study, we aimed to quantitate the frequency of queries by women seeking information in an internet search engine after missing 1 or more doses of an OC and their further queries on emergency contraception, abortion, and pregnancy.

## Methods

We extracted all English-language search queries submitted to Bing by users in the United States during 2018. Bing’s market share in the United States was estimated at 25% [27]. It is estimated that Bing users are a representative sample of the US population [28]. Data for each search query included an anonymous user identifier, time and date of the search, and search text. User gender and age groups, reported when users registered to Bing, were available for a subset of the users.

The text of the queries was used to filter the queries into 1 of 5 classes (Textbox 1).

Using the pregnancy queries, we calculated the first day of the last menstrual period (LMP) for women who reported pregnancy by subtracting the number of weeks reported in their queries from the date of the query.

The likelihood of pregnancy was calculated as the percentage of women who queried for a missed OC dose and later queried for a week of pregnancy. The empirical likelihood to query about each week of pregnancy was calculated as the fraction of queries, which mentioned a specific week of pregnancy. To compensate for the finite data period, each user who reported a missed dose was assigned a weight relative to the likelihood for querying about pregnancy, relative to the date of reporting about the missed dose. For example, if a user asked about a missed dose in January, they would be given a weight of 1 since the full term of pregnancy was within the data period. Conversely, a user who queried during December was given a low weight, since they could only query for the first few weeks of pregnancy.

Statistical analysis was conducted using MATLAB 9.7 with the statistical toolbox version 11.6. This study was approved by the Behavioral Sciences Research Ethics Committee of the Technion.

**Classes of search queries of users.**

1. Missed OC queries: queries which contained the words “miss,” “skip,” or “forgot” (and their variations, eg, forgotten) and the name of an OC (both brand and generic), or the phrase “minipill,” “birth control,” or “contraceptive.”
2. Miscarriage queries: queries which contained the words “after miscarriage,” “post miscarriage,” or “I had a miscarriage.”
3. Abortion queries: queries which contained the word “abortion,” excluding queries referring to specific legislation, abortion debates, or celebrities who had an abortion.
4. Emergency contraceptives: queries which contained the words “plan b” or “morning after pill,” excluding queries referring to specific legislation, abortion debates, or celebrities who had an abortion.
5. Pregnancy queries: queries which contained the words “week” and “pregnancy” (eg, “what to expect on week 21 of pregnancy”). Past studies have shown that such queries have a high specificity for actual pregnancy [29].

## Results

### Accuracy of Identification

We validated the identification of relevant queries by manually inspecting the 20 most common queries of each of the 5 classes for whether they could be interpreted as relevant to the topic. For example, queries regarding general news on OC use were deemed irrelevant to the research topic and were excluded from the study. Two of the authors independently reviewed these queries and marked them for relevancy. Kappa agreement between the labelers was, on average, 0.866 (n=5). Considering only the queries that both labelers agreed to be relevant, 75% of missed OC queries; 100% of pregnancy, miscarriage, and abortion queries; and 80% of emergency OC queries were correctly identified.

### Missed OC Queries

In 2018, 26,395 Bing users in the United States queried about missing OC pills. Of these, 60.8% did not mention the type of OC they missed, 20.9% mentioned a combined OC brand, and 21.7% mentioned a progestin-only OC brand or minipill (a total number of users is greater than 100% since a minority of users mentioned multiple types). Of users who mentioned the number of missed doses, 21.4% mentioned forgetting 1 dose; 6.3% forgot 2 doses; 3.4% forgot 3 doses; and 1.8% forgot 4 or more doses. Other users did not mention the specific number of doses they forgot, and their queries did not directly indicate this information.

There was a weak positive correlation (ρ=0.07, *P<*.001) between user age and the number of missing doses.

**Figure 1:**
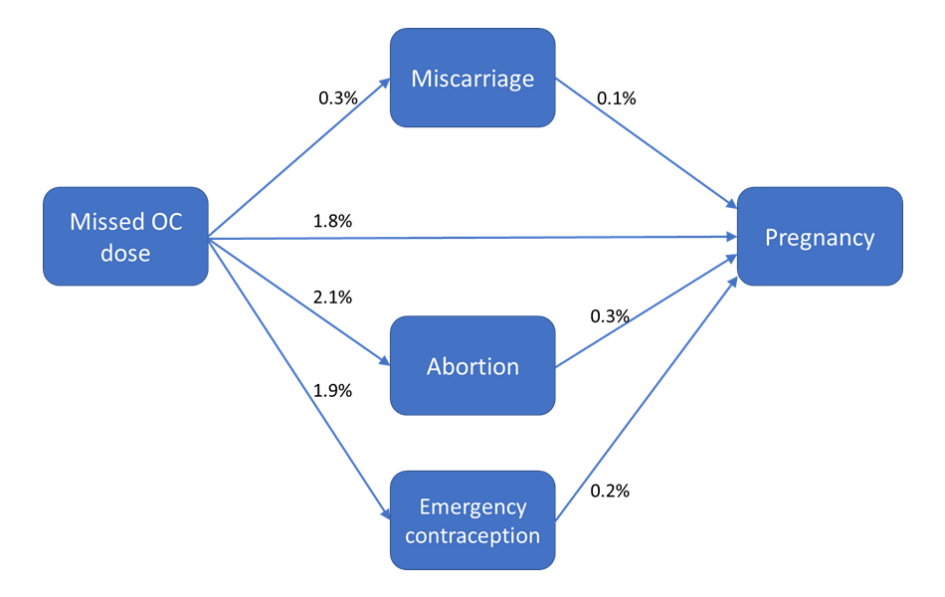
Percentage of users who asked about missed OC doses and then asked about miscarriage, pregnancy (as indicated by a query for the week of pregnancy), abortion, and emergency contraception as well as the percentage of users who asked about missed OC doses, followed by a query about miscarriage, abortion, or emergency contraceptive, before querying about the week of pregnancy.

Figure 1 shows the percentage of users who asked about a missing OC dose and then queried about abortion, miscarriage, emergency contraception, or a week of pregnancy (the latter indicating that they most probably were pregnant at a later time). Furthermore, it shows how many of the users who query for the first 3 classes followed with queries for a week of pregnancy.

**Figure 2:**
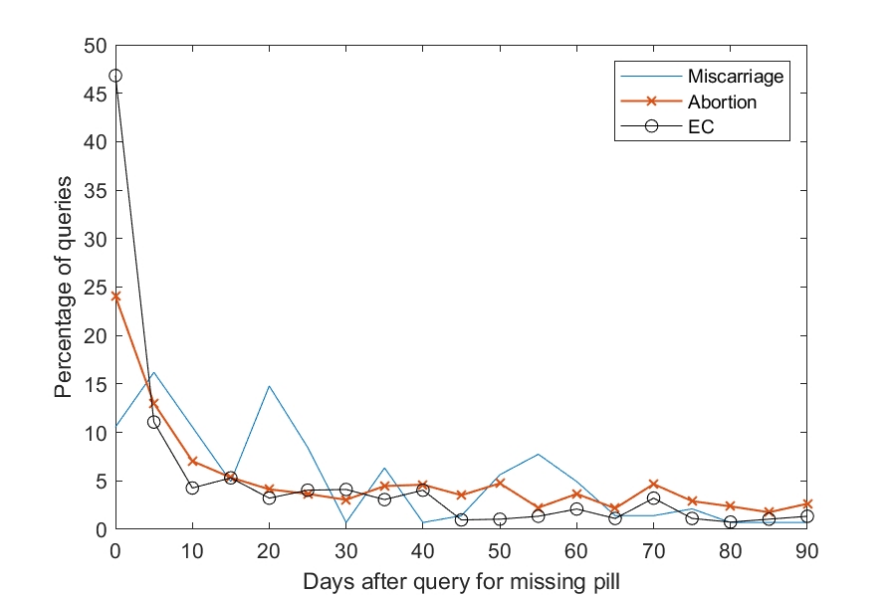
Distribution of the number of days between queries for a missed OC dose and for miscarriage, abortion, and emergency contraceptive.

The majority of the queries for these 3 classes occur within a few days of the missed OC dose query (Figure 2). The distribution of queries that were made within 24 hours after a query about missing an OC was as follows: 7% of miscarriage queries, 20% of abortion queries, and 37% of emergency contraceptive queries. The median query time after a query about missing an OC was 35 days for miscarriage queries, 28 days for abortion queries, and 11 days for emergency contraceptive queries.

### Unintended Pregnancies Following a Missed OC Query

Each user who reported a missed dose was assigned a weight relative to the likelihood for querying about pregnancy, relative to the date of reporting the missed dose (see Methods). The weighted percentage of women who queried for pregnancy week following a query for a missed pill was 4.7%. Only 19% of these queries could be timed to the cycle where the same women had queried about missing a pill. We calculated the inferred LMP from each of these queries. Among the 3130 women who queried for pregnancy at different weeks of gestation, there was a good consistency between the calculated LMP for recurrent queries, and the median number of days between the inferred LMP was 4 days.

Some women mentioned in their query that they missed a placebo pill. For those, the rate of pregnancy was 0.2% versus 4.5% for the general population. The weighted rate of pregnancy for women who mentioned taking a minipill was 8.7% versus 4.7% for other medications.

From queries on the week of pregnancy, we calculated the first day of the last cycle. The average day of conception was 14.4 days (SD 7.2) after the first day of the cycle.

The weighted percentage of females who queried for pregnancy following a query for an unspecified number of missed pills was 4.7%. The weighted pregnancy rates of females who queried for 2 or more missing doses was 5.1% (ie, 8.5% higher).

### Age Distribution of Users Searching for Information on Missed OC

**Figure 3:**
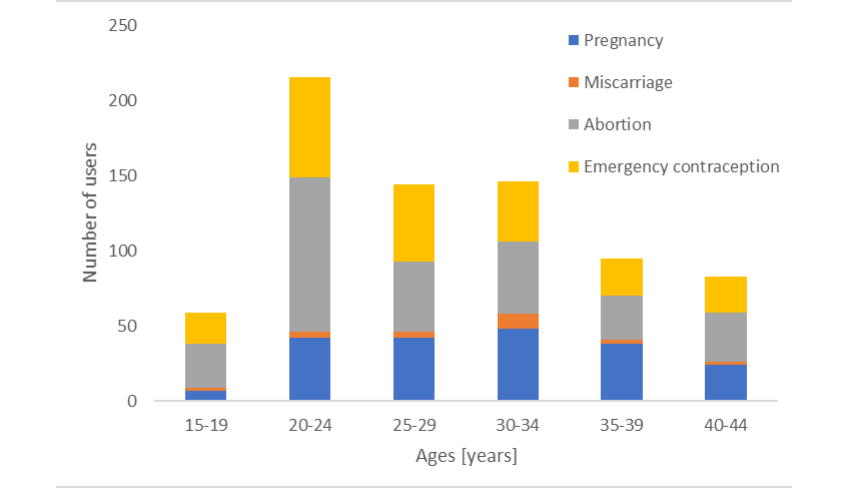
Number of users by age group who asked about missed OC doses and each of the other query categories.

The average age of users who queried for a missing dose was 32 (SD 12) years (mode=20 years). Figure 3 shows the distribution by age of users who asked about missed OC dose and then each of the other query types. Figure 3 demonstrates that users under the age of 30 years are more likely to ask about abortion (1.5 times) and emergency contraception (1.7 times) (chi-square *P*<.001 for both), while users at ages of 30-34 years are more likely to query about pregnancy (2.1 times) and miscarriage (5.4 times) (chi-square *P*<.001 for both).

## Discussion

The study identified a myriad of women who queried missing an OC pill. Young women are more likely to miss a dose or multiple doses and to search for information on emergency contraceptives and abortion. Following a missed OC query, abortion and miscarriage queries are common, peaking in the first few days after the missed OC.

A small but not insignificant percentage of women querying for information after missing an OC dose eventually get pregnant in the month of query. As expected, the likelihood of pregnancy after missing a placebo pill is practically zero, and the likelihood of pregnancy after missing a progestin-only pill is double the risk of missing a dose of combined OC.

A majority of people occasionally forget to take a dose of a chronic medication, and studies have found that about 1 in 2 young women miss a dose of OC regularly [15-18]. Our data indicate that some women missing a dose of OC did not obtain sufficient information from their health care providers on the consequences of missing a pill, and thus, they resort to search the relevant information on the internet. Search for emergency contraception, which peaks in the first few days after querying about missing an OC dose, is an additional sign of inadequate information supplied by the health care provider.

Queries about abortion and miscarriage peak in the first few days, following a query about forgetting an OC dose. Many of these queries are most likely not an indication that the woman has confirmed being pregnant but a sign that she is very worried of being pregnant and thinking ahead about her further options of dealing with a possible unwanted pregnancy.

Our results also show that only one-third of queries about emergency contraception occur within the first 24 hours after the missed OC query. This, together with the fact that the median time between the queries is 11 days, indicates that many women are finding information on emergency contraception too late for it to be useful.

The strength of this study is access to the moment of uncertainty and information-seeking behavior of a large number of women missing a dose of OC. The limitations are absence of information on missed OC doses in other months or further doses in the same month. Pregnancy or its absence cannot be comprehensively confirmed.

The main implication of our results is reinforcement of the importance of providing comprehensive written information and directing patients to reliable information resources on missed pills. Most women are more familiar with what to do when one misses a pill, but they lack the knowledge of what to do when missing more than 1 OC dose [19]. Therefore, it is important to provide instructions for when a woman misses more than 1 OC pill in a package.

As young women are more likely to miss a pill, it may be more effective to offer women in these age groups contraceptive alternatives that do not depend on daily compliance. Health care providers should establish special counseling methods for young women who choose OCs and make sure that the instructions are understood by those young women. All OC users should also be provided with information on emergency contraceptive options and rapid access to an emergency contraception in case it is needed due to missed pills and unprotected intercourse. As young people often turn to internet search engines for health advice, health providers could comply with the WHO guidelines for contraceptive use by not only supplying information during people’s visit to the clinic but also adding detailed information on preventing pregnancy in case of missed OC doses to their websites or as part of a mobile app that is given to patients at the initial contraceptive consultation [21]. In addition, it is important that resources with accurate information be provided by search engines when people search for “missed pill” or any of the terms identified and used for this study. Better information can lead to better planning of contraceptive use when a pill is missed, reducing the risk of pregnancy and preventing stress and anxiety related to the fear of an unwanted pregnancy.

## Abbreviations

LMP: last menstrual period
OC: oral contraceptive
WHO: World Health Organization
